# Patient-Planetary Health Co-benefit Prescribing: Emerging Considerations for Health Policy and Health Professional Practice

**DOI:** 10.3389/fpubh.2021.678545

**Published:** 2021-04-30

**Authors:** Nicole Redvers

**Affiliations:** ^1^Department of Family & Community Medicine, University of North Dakota School of Medicine & Health Sciences, Grand Forks, ND, United States; ^2^Nuffield Department of Primary Care Health Sciences, University of Oxford, Oxford, United Kingdom

**Keywords:** planetary health, co-benefits, health professionals, climate change, sustainable healthcare, prescribing practices, Indigenous knowledges

## Abstract

In addition to the importance of fostering and developing measures for better health-system resilience globally from the effects of climate change, there have been increasing calls for health professionals, as well as public health and medical education systems, to become partners in climate change mitigation efforts. Direct clinical practice considerations, however, have not been adequately fostered equitably across all regions with an often-confusing array of practice areas within planetary health and sustainable healthcare. This article calls for a more coordinated effort within clinical practice spaces given the urgency of global environmental change, while also taking lessons from Indigenous traditional knowledge systems—a viewpoint that is rarely heard from or prioritized in public health or medicine. Simpler and more coordinated messaging in efforts to improve patient and planetary health are needed. The creation of unifying terminology within planetary health-rooted clinical and public health practice has been proposed with the potential to bring forth dialogue between and within disciplinary offshoots and public health advocacy efforts, and within clinical and health-system policy spaces.

## Introduction

Planetary health is a field focused on characterizing the linkages between human-caused disruptions of Earth's natural systems (e.g., climate change, deforestation, pollution) and the resulting impacts on public health (1). The field of planetary health aims to develop and evaluate evidence-based solutions to safeguard an equitable, sustainable, and healthy world (2). This aim is platformed on a sense of urgency underscored in a recent Intergovernmental Panel on Climate Change (IPCC) special report outlining the need to make drastic cuts in greenhouse gas emissions by 2030 (by about 45% from 2010 levels) in order to prevent temperature rises exceeding 1.5 degrees Celsius above pre-industrial levels (3). With this, there is a proposed stronger need for climate action in wider spheres of influence, which includes public health-system structures and clinical practice. Ultimately, the contributions toward an action-orientated road map to net zero greenhouse gas emissions within healthcare needs to align with current planetary health and sustainability targets globally.

If the global healthcare sector were its own country, it would be the 5th largest greenhouse gas emitter on the planet (4). Acknowledging the clear impacts between various interconnected global environmental changes [e.g., global warming, pollution, biodiversity loss (see Table 1 for important definitions)] in the health of humans and other species, the current Director General of the World Health Organization, Tedros Adhanom Ghebreyesus has stated that, “Health sector facilities are the operational heart of service delivery, protecting health, treating patients, and saving lives. Yet health sector facilities are also a source of carbon emissions, contributing to climate change. Places of healing should be leading the way, not contributing to the burden of disease” (4). The Sustainable development goals (SDGs), adopted by all United Nations Member States in 2015, also ensures a spotlight on the need for improving health and reducing inequalities, while also tackling climate change and preserving biodiversity (12). Therefore, there is a clear need to bring together health and sustainable development using strategies that reduce greenhouse gas emissions, pollution, and deforestation (13).

**Table 1:** Important terminology used in the field of planetary health.

**Terminology**	**Definition**
Planetary Health (Western)	A field focused on characterizing the linkages between human-caused disruptions of Earth's natural systems and the resulting impacts on public health (1).
Planetary Health (Indigenous)	Planetary health as a “field” is primarily a Western construct as Indigenous Traditional Knowledge systems have no clear separation between the health of the planet and the health of self or that of the community and the ecosystem at large (5). This means that the meaning and applications of planetary health are directly rooted in community values based on protocols for living in harmony with all that have existed for thousands of years (6).
Climate Change	A change in climate that is attributed directly or indirectly to human activity that alters the composition of the global atmosphere and that, in addition to natural climate variability, is observed over comparable time periods (7).
Global Warming	The long-term heating of Earth's climate system observed since the pre-industrial period due to human activities, primarily fossil fuel burning, which increases heat-trapping greenhouse gas levels in Earth's atmosphere (8).
Global Environmental Change (GEC)	Large-scale and global environmental changes include climate change, stratospheric ozone depletion, changes in ecosystems due to loss of biodiversity, changes in hydrological systems and the supplies of freshwater, land degradation, urbanization, and stresses on food-producing systems (9).
Biodiversity Loss	The decrease in the variety of life on Earth in all its forms (i.e., the diversity within species, between species and of ecosystems) (10). One million species out of an estimated total of eight million species are threatened with extinction, many within decades (10).
Co-benefits	The potentially large and diverse range of collateral benefits that can be associated with climate change mitigation policies in addition to the benefits derived from directly avoiding climate impacts (11).

In 2015, the World Health Organization (WHO) published an operational framework for building climate resilient health systems (14). The focus of the report was to enable health systems globally to be better able to anticipate, prevent, prepare for, and manage climate-related health risks (14). Despite the importance of fostering and developing measures to ensure health-system resilience globally from the effects of climate change (i.e., adaptation), there have been increasing calls for health professionals, as well as public health and medical education systems, to become partners in climate change mitigation efforts (1, 15, 16). Despite this call to action for health-professional involvement in mitigation efforts, there is evidence demonstrating that in some regions clinicians may not actually feel comfortable counseling patients specifically around climate change impacts and health (64% percent of primary care physicians in one US state believe climate change was affecting their patients' health, whereas only 17% were comfortable counseling patients about climate change and health) (17). Direct clinical practice considerations have therefore not been adequately fostered at the health provider or institutional level, with current policy planning efforts for developing meaningful mitigation and adaptation measures often focusing on interactions and impacts outside of the clinical exam room. Although warranted, if health professionals are to be called to action in the fast-evolving climate change crisis, more scholarly, clinical, and policy attention is urgently needed in how to operationalize this call.

To date, there have been a number of actions specifically proposed that clinicians can take in promoting individual and planetary health within their own practices and communities (1, 18). These stated planetary health actions stem from climate adaptation measures through the lens of preparedness and responsiveness, to global environmental change mitigation measures through advising patients directly within clinical care on potential co-benefit actions for people and the environment (see Table 2). Additionally, there has been increasing focus on the “Why? What? And How? of educating for environmentally sustainable healthcare education” (29, 30), as well as the push to re-define quality improvement (QI) in healthcare to include environmental sustainability (31, 32). Even specialty-specific environmental sustainability best practices are ongoing (33, 34), with regional organizations such as the Center for Sustainable Healthcare leading the way in offering strategic input, consultancy, and training in sustainable healthcare practice within the UK specifically as well as abroad (35). There are also continually evolving academic and practice movements within environmental public health, One Health, EcoHealth, and of course Planetary Health. Each approach to tackling the urgency of climate change and biodiversity loss has its strengths and opportunities; however, for clinicians, the multitude of terminologies and areas of discourse do not always translate down to the clinical exam room. With this, there is an important need for synergy among any proposed clinical approaches that seek to provide benefit to both the patient and the planet.

**Table 2:** Summary of some of the existing patient-planetary co-benefit actions that have been proposed [partially adapted from WONCA Working Party on the Environment (1)].

**Co-benefits**	**Description**
Food choices	A transition to a more sustainable plant-based diet—rich in fruits, vegetables, nuts, and legumes—can reduce the environmental footprint of agriculture, as recently highlighted by the EAT-Lancet Commission (19, 20).
Active transport	Forms of transport that involve physical activity, such as cycling and walking, have the dual benefit of reducing emissions and protecting against multiple diseases (13).
Reproductive health	Ensuring universal access to reproductive healthcare can improve both maternal and child health and limit population growth by reducing unwanted pregnancies (21).
Connecting within nature	Finding ways to spend more time outside in nature—including in green space in cities—can have benefits for physical and mental health (22) and increase a sense of stewardship for our natural environment (23, 24).
Engaging in community	Fostering social connectedness through community building not only results in mental-health benefits, but can also help build the social capital necessary for collective action (25). Connecting with those around us is thought to be particularly effective for planetary health when mobilizing around a common goal, such as bringing more green space, bike lanes, composting services, or farmers' markets to our communities (1)
Sustainable drug prescribing	Eco-directed Sustainable Drug Prescribing (EDSP) has been proposed to prevent the adverse effects of some active pharmaceutical ingredients (APIs) in the environment (26). Other medications have been highlighted for their global warming potential (GWP) with statements being issued to inform clinicians on potential alternatives (27).
Preventative medicine	With an increase in the overall global population as well as the aged, the carbon footprint of healthcare is not improving, and the complexity of diagnostic and treatment methods used is increasing (28). Disease prevention strategies are important for patients as well as for reducing the intensity of the high carbon care required (28).

### Patient-Planetary Health Co-benefit Prescribing

**Figure 1:**
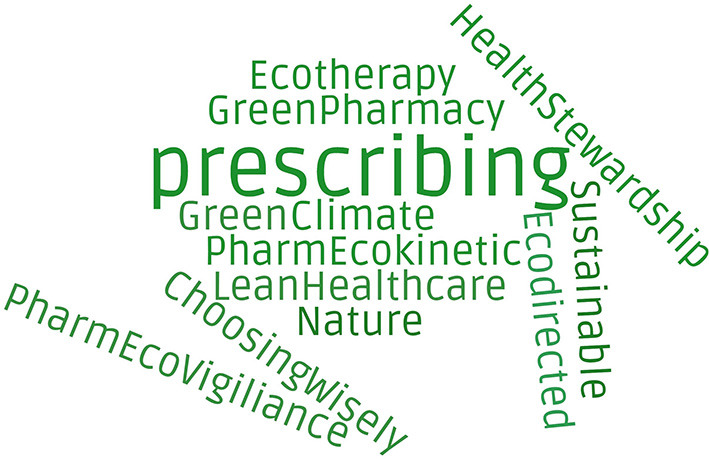
Some examples of the multiple and often independently evolving prescribing terminologies and practice areas that seek to improve the health of people and planet.

The term “co-benefits” has increasingly been used in climate change and planetary health discourse. Instead of considering climate change specifically in isolation for example, a co-benefit approach considers how climate change mitigation initiatives can also advance other policy goals (36), while also leading to improvements in health (37). This fits in with the aims of planetary health, which is to develop and evaluate evidence-based solutions to safeguard an equitable, sustainable, and healthy world (2). Operationalized clinically, a co-benefit approach would seek to consider both the individual's and the planet's health in the context of the medical advice given (1). What is not explicit in the co-benefit framing however is the often nuanced uneven prioritization of people over planet in policy and implementation. In considering a new framework for defining co-benefits, while taking into consideration the multiple evolving terminologies and practice areas that seek to improve the health of people and planet (see Figure 1), a synergistic and interconnected perspective is needed that highlights healthy patients and a healthy planet without hierarchy, terminology dissonance, and overall confusion. Lessons can be gleaned in this regard from Indigenous knowledge systems as presented below.

Indigenous languages worldwide, for example, are completely and utterly based and rooted within relationships to the land (38). The process of putting words and terminologies together is not without careful thought to their relationships with each other, and the symbolisms, interpretations, and actions that could come from their use. This is exemplified by the tight connection between language and Indigenous knowledges as it relates to biodiversity (39). In considering this, how we use language can be a powerful tool for both awareness and implementation processes within and outside of healthcare (40).

In the current efforts to create climate-friendly clinical prescriptions, or clinical consults with climate change in mind, we are automatically framing our work through the lens of an issue or a problem to be solved *outside* of ourselves (i.e., climate change). This is in direct opposition to the Indigenous worldview in which everything is entirely interconnected (41), and demonstrated additionally by the Indigenous conceptualization of planetary health seen in Table 1. With environmentally sustainable clinical practices developing in response to a “state” (i.e., the climate crisis), as opposed to the focused recognition of the need for “ongoing” interconnected relationship building between patient and planet (i.e., ongoing stewardship vs. a situation-based action), we mechanize our process of clinical delivery against a deficit state. Patient-planetary health considerations and therefore discourse should be framed in perpetuity from a strengths-based perspective regardless of the state of the world's environment and climate. Climate change or not, it is important to understand that environmental values are human values, that our planet is a responsive relative, and that public health and healthcare stewardship can only be truly effective through an interconnected worldview that prioritizes ongoing relationships instead of reactive elements and current states. Health professionals have a moral responsibility to deeply consider their “professional role to promote public health and address threats to psychological and physical welfare” with the climate and biodiversity-loss crisis lying “within the moral remit of medicine” (42); however, this should not be bounded by crisis alone.

The joining of “patient-planetary health” therefore is purposeful in that the grammatical hyphen itself is connecting the two words together, subtly elucidating their direct interconnection. The joining of the terms also seeks to remove any hierarchical inclination of one focus over the other, instead ensuring the view is always on both elements equitably in considering a co-benefit perspective. “Patient-planetary health co-benefit prescribing” would therefore be overarching terminology to denote any and all *prescribing* practices that have co-benefits for both patient and planetary health. Patient-planetary health co-benefit prescribing may therefore provide a potential umbrella lens for how we might consider co-benefit practices within public health and healthcare spaces, with room to allow flexibility under the heading itself. Putting heuristics aside, the creation of unifying terminology within planetary health–rooted clinical and public health practice spaces has the potential to bring forth dialogue between and within disciplinary offshoots and advocacy efforts, and within public health and health system policy spaces. Consistency in language, coordinated efforts and messaging across public health, health systems, and policy spaces have been exemplified within other areas of prescribing practice, including the area of antimicrobial stewardship, which required mobilizing transdisciplinary behavioral approaches across multiple regions (43). Lessons can be learned from other complex efforts to improve patient and planetary health.

With a growing multitude of international efforts, we need clearer messaging for our care providers and health-system leaders alongside our push for planetary health aware, climate-responsive health systems. Patient-planetary health co-benefit prescribing practices (e.g., sustainable drug prescribing, nature prescribing, planetary friendly diets, etc.), may offer one way to drive change from within clinical care systems with the path made clearer by unifying the confusing array of sustainability-practice terminologies under one umbrella heading and a field of practice (i.e., planetary health). Patient-planetary health co-benefit prescribing may also itself fall under greater planetary healthcare frameworks (28), or sustainable quality improvement processes (31, 32) that also encompass sustainability and stewardship needs *outside* of the patient care room.

An overarching systems approach to implementing planetary healthcare frameworks within public health and healthcare is also needed (28, 44). Success may be more likely by specifically focusing on leveraging existing relationships between organizations (planetary health, EcoHealth, One Health, environmental public health, etc.), and clinical practice approaches (green prescribing, climate prescribing, etc.) for greater overall dissipation and impact (i.e., planetary health aware, climate-responsive health systems down to the patient care level). Given the complexity parameters surrounding both health systems and global environmental change, emergent areas of patient-planetary health co-benefit practice points can optimally be further refined, organized, and deliberate in their developing application with patients, institutions, and with health policy planning. Due to the variances in health-system delivery, political structures, and payment models globally, specific locally appropriate adaptations and policy considerations will likely be needed under the patient-planetary health co-benefit umbrella. Behavioral systems approaches that take into account individual and policy level drivers (45) may also be applicable and potentially leveraged in the process of further developing clinical and public health practice guidelines given the importance of clinician behavior change in any implementation process (45, 46).

Notwithstanding, this proposed effort and dialogue also attempts to break down traditional public health and healthcare silos, including within many current environmental healthcare sustainability spaces, which have historically and contemporarily minimized the voices and knowledge systems of Black, Indigenous, and People of Color (BIPOC) communities. For example, given “the colonial legacy, Indigenous people, despite their essential knowledge systems and abilities, still face many barriers in terms of access to safe spaces and presence, but also in the ability to take on active leadership roles in health professions education,” and therefore additionally in education for sustainable healthcare (41). Holistic discourse is needed within planetary health broadly that amplifies lessons learned from *Indigenous Peoples*' traditional medicine systems and their long-standing sustainable models of health delivery (47).

The training of our Indigenous healers through traditional Indigenous ways of learning is centered around a values-based system that reflects the interconnectedness of all things. These values promote and uplift shared stewardship goals through a set of natural laws derived from the land on which the training takes place. There has so far been a lost opportunity for potential learning from the long histories of Indigenous healing and environmentally sustainable medicine traditions around the world that could inform sustainable healthcare practices. These healing knowledge systems are based on the notion of collective responsibility for health and wellness without hierarchy that includes innate notions of environmental stewardship (41).

Knowledge translation and intercultural dialogue between Western and Indigenous health systems (48, 49), as well as evolving scholarship (50, 51), expanding integrative health delivery systems (52–54), and discussions around the ethical basis for medical pluralism (55, 56) is on the rise. Despite differing worldviews between biomedical clinical settings and Indigenous traditional knowledge settings, we are at a clear interface in time where our ability to sustain ourselves on this planet is in question. Indigenous traditional knowledge is deep, evidenced-informed, resilient, and time tested, yet it does not come without Indigenous Peoples themselves. Established international agreements that serve to protect Indigenous Peoples and their knowledges, and the proactive protection of intellectual property and data sovereignty rights must be front and center to avoid the further perpetuation of inequities and discrimination of Indigenous Peoples (57). Moving forward “in a good way” (58) will require the elevation and amplification of Indigenous voices within healthcare spaces, including within planetary health and sustainability spaces (41).

## Conclusion

Simpler and more coordinated messaging in health system efforts to improve patient and planetary health are needed. The creation of unifying terminology within planetary health–rooted clinical and public health practice spaces has been proposed with the potential to bring forth dialogue between and within disciplinary offshoots and public-health advocacy efforts, and within clinical and health-system policy spaces. The critical need and purpose for defining patient-planetary health co-benefit prescribing frameworks will be seen to have been historically rooted in the climate change crisis; however, it will hopefully be predicated on the innate understanding that patients, their communities, and the planet are locked into an interconnected web of relationships that crosses all boundaries of systems, policy, and practice. An exemplified interprofessional planetary health pledge has recently been proposed (59) that embodies the importance of epistemological pluralism and diversity in action, while also recognizing the interconnected entanglement between individual, community, and planetary health. We are ultimately stronger in our cultural and knowledge diversity, including in the ways we practice public health and medicine. Gone are the times when focusing solely on human-centric approaches to health will make us and our communities well. In further considering the current dual climate-biodiversity loss crisis and the pandemic crisis, human-centric approaches to health and well-being was never really an option for us—we have fallen too far away from our healing relationship and interdependence within nature. This nature disconnect will only grow unless we consider transformative changes inside and outside public health and healthcare spaces.

“*…a key argument put forward by Indigenous knowledge systems is that health, and ill-health, emerges out of both the nature of the environment within which the individual lives and the individual's connection with that environment.”* (60)

## Data Availability Statement

The original contributions presented in the study are included in the article/supplementary material, further inquiries can be directed to the Corresponding Author.

## Author Contributions

The author confirms being the sole contributor of this work and has approved it for publication.

## Conflict of Interest

The author declares that the research was conducted in the absence of any commercial or financial relationships that could be construed as a potential conflict of interest.
